# A Thai version of the Michigan hand questionnaire (Thai MHQ): an investigation of the psychometric properties

**DOI:** 10.1186/s12955-020-01548-0

**Published:** 2020-09-22

**Authors:** Pichitchai Atthakomol, Worapaka Manosroi, Saran Sanguanrungsirikul, Siraphop Punoppamas, Sirapat Benjachaya, Siam Tongprasert, Tinakon Wongpakaran

**Affiliations:** 1 Department of Orthopaedics, Faculty of Medicine, Chiang Mai University, Chiang Mai, Thailand; 2 Musculoskeletal Science and Translational Research Center, Chiang Mai University, Chiang Mai, Thailand; 3 Division of Endocrinology, Department of Internal Medicine, Faculty of Medicine, Chiang Mai University, Chiang Mai, Thailand; 4 Department of Rehabilitation Medicine, Faculty of Medicine, Chiang Mai University, Chiang Mai, Thailand; 5 Department of Psychiatry, Faculty of Medicine, Chiang Mai University, Chiang Mai, Thailand

**Keywords:** Thai MHQ, Reliability, Validity, Responsiveness

## Abstract

**Background:**

The Michigan Hand Questionnaire (MHQ) is widely used to assess the hand/wrist conditions. We translated the original version into Thai (Thai MHQ) and evaluated its psychometric properties.

**Methods:**

After receiving permission, the original MHQ was translated and cross-culturally adapted to Thai following standard guidelines. Two hundred and seventeen patients who had hand/wrist injuries or disorders were included in the study. Internal consistency was evaluated using Cronbach’s alpha. Test-retest reliability was analyzed using the intraclass correlation coefficient (ICC). Spearman’s rank correlation among the subscales of Thai MHQ, Thai DASH and Thai EQ-5D-5L and also confirmatory factor analysis (CFA) were used to explore construct validity. The standardized response mean (SRM) was used to evaluate the responsiveness of the Thai MHQ.

**Results:**

All subscales showed an acceptable Cronbach’s alpha (0.79–0.98). The test-retest reliability of each subscale was good (ICC = 0.83–0.95). In related dimensions, strong correlation was demonstrated between the Activities of daily living subscale of the Thai MHQ and the Common activities subscale in the Thai DASH (*r* = 0.77, *P* < 0.0001). For unrelated dimensions, a weak correlation was found between the Aesthetics subscale in the Thai MHQ and the Mobility subscale in the Thai EQ-5D-5L (*r* = − 0.13, *P* = 0.05). The Thai MHQ had strong correlation with Thai DASH (*r* = − 0.79, *P* < 0.0001) and Thai EQ-5D-5L (*r* = 0.63, *P* < 0.0001). CFA showed that the 6-factor model demonstrated an acceptable fit to the data. The SRM of the Thai MHQ was 0.78, indicating relatively large responsiveness. The MIC of Thai MHQ using distribution methods (SEM) was 5.2.

**Conclusions:**

The Thai MHQ provides adequate internal consistency in all subscales as well as good construct validity and reliability for Thai patients and a relatively large standardized response mean at 2 months after treatment.

## Background

Hand and wrist are common areas for upper extremity injuries [1–6]. To evaluate hand/wrist function and severity in the initial stage or after treatment, both clinicians and researchers need standard tools which have high reliability, validity and the ability to detect clinically important changes overtime [7, 8]. During the past two decades, patient-rated outcome measurements have increasingly been used in healthcare systems. Benefits of these instruments include that they can infer the patient’s perspective on many aspects which affect outcomes, while traditional outcome measurements are assessed based on the views of clinical examiner [9–12].

The Michigan Hand Outcomes Questionnaire (MHQ) is one of the widely used patient-rated outcome measurement instruments for patients with hand disorders. This questionnaire is the subjective assessment for musculoskeletal, neurological or nonspecific pathology which providing the ordinal data type according to The World Health Organization International Classification on Functioning Disability and Health (WHO ICF) summary [13]. The original version was developed in 1998 by Chung et al. [10]. Among the benefits of this instrument are its specificity to hand/wrist injuries or disorders, the capacity to evaluate impairment both individually for each hand as well as for both hands together and the ability to assess more aspects including overall hand function, activities of daily living, pain, work performance, satisfaction and specially in aesthetic compared to Disabilities of Arm, Shoulder and Hand (DASH) questionnaire [10, 11, 14]. Previous studies have also reported that the MHQ was more responsive than DASH in patients with traumatic hand injury, carpal tunnel syndrome or wrist pain [15, 16].

Evaluations of psychometric properties of the MHQ have documented high internal consistency and test-retest reliability [10, 14, 17–19]. Furthermore, this tool has been proven to be valid and has demonstrated a high standardized response mean (SRM) in many types of hand/wrist injuries and disorders [10, 14, 18–22]. The MHQ has been translated and cross-culturally adapted into many languages including Turkish [23], Brazilian [24], Korean [25], Japanese [26], German [27], Persian [28], Polish [29], French [30], Finnish [31] and Thai [32].

Presently, what is lacking is a hand/wrist outcome measurement which is reliable, valid and responsive specifically for Thai patients who have hand/wrist injuries or disorders. Although a Thai version of MHQ has recently been reported by Dhippayom et al., that version has some limitations. The study was conducted with a small sample size (30 patients) and demonstrated a relatively low statistical power (0.51). Additionally, factor analysis and responsiveness evaluations have not been reported [32].

The aims of this study were to create a Thai version of the original MHQ, including translation and cross-cultural adaptation, and to investigate the psychometric properties of the Thai version including reliability, validity and responsiveness.

## Methods

The first of two stages in this study was the translation and cross-cultural adaptation of the original MHQ to create a Thai version. The second step was to explore the psychometric properties of the Thai MHQ, including reliability, validity and responsiveness. We hypothesized that the Thai version could maintain the reliability, validity and responsiveness of other versions of MHQ. This observational study was approved by the local institutional research ethics committee.

### The MHQ questionnaire

The Michigan Hand Outcomes Questionnaire (MHQ) consists of specific questions in 6 subscales including overall hand function (5 items each for left and right hand), activities of daily living (5 items each for left and right hand, 7 items for both hands); work performance (5 items); pain (5 items each for left and right hand); aesthetics (4 items each for left and right hand); and satisfaction with hand function (6 duplicate items each for left and right hand) [10]. The reliability, validity, and responsiveness of the MHQ has been investigated for many conditions of hand/wrist disorders and has been translated into many languages [10, 14–16, 19–25, 27–32]. Raw scores are converted to scores with a range of 0 to 100. For the Pain subscale, higher scores indicate more pain. For the other subscales, higher scores indicate better hand performance.

### Translation and cross-cultural adaptation

The authors requested permission from Chung et al. to translate the original version into Thai. Translation and cross-cultural adaptation was performed following the recommendations of Beaton et al. which consist of 5 stages as described below [33].

- Stage 1. Forward translation. Two bilingual translators (a Thai medical student and an academic English language instructor) independently translated the original MHQ into Thai versions (T1 and T2) [10].
- Stage 2. Synthesis. The two translators discussed differences point by point and created a synthesis of the T1 and T2 versions (T-12). The differences were also discussed with an Orthopaedic hand specialist to arrive at a consensus.
- Stage 3. Back translation. The T-12 version was back translated into English (BT1 and BT2 versions) by two bilingual English/Thai speakers (English language lecturers) to ensure that the concept of the original version was maintained.
- Stage 4. Review by an expert committee on hand surgery and measurement development. An expert committee, composed of one hand surgeon, one rehabilitation specialist, one psychiatrist and two linguistics experts, reviewed and consolidated the pre-final version of Thai MHQ from all the translated versions (T1, T2, T12, BT1, BT2).
- Stage 5. Field testing of the pre-final version. Field testing was conducted with 15 healthy volunteers and 15 patients who had hand/wrist injuries. All were native Thai speakers who were fluent in reading, writing and understanding the Thai language. They were requested to examine all instructions and items for item-objective congruence (IOC) and to provide suggestions. If any item demonstrated an IOC value less than 0.5, that item was reconstructed and reevaluated until the IOC value reached at least 0.5. Finally, the pre-final version of the Thai MHQ was translated back into English and sent to Chung et al. for approval.

### Psychometric testing of the Thai MHQ

Internal consistency, construct validity, factor analysis, test-retest reliability and responsiveness of Thai MHQ were evaluated in Thai patients who were enrolled prospectively at the Hand Outpatient Clinic of Academic University Hospital from December 2018 to December 2019. Inclusion criteria were patients who had hand/wrist musculoskeletal disorders, used Thai as their first language and had the ability to read and understand Thai, age between 15 and 70 years and had the ability to complete the questionnaire without major assistance. Patients who had a musculoskeletal disorder above wrist level, an active cerebral disorder or communication problems were excluded. Since there were many types of hand and wrist injuries or disorders, the treatments might include both conservative and operative treatments. The follow-up schedule to evaluate responsiveness was at least 4 weeks after treatment depending on the diagnosis of each patient. This study adhered to the COnsensus-based Standards for the selection of health Measurement Instruments (COSMIN) checklist [34].

After giving informed consent, patients were enrolled in study. Patient demographic data recorded include age, sex, dominant hand, injured hand, diagnosis and time taken to complete the questionnaire.

### Internal consistency

Internal consistency is the degree of correlation between items measuring the same outcome. Cronbach’s alpha was used to measure the internal consistency of each subscale and the total score of Thai MHQ. Values can range from 0 to 1, with higher scores indicating greater interrelatedness between items. Values of 0.70 or higher are considered to be adequate [35].

### Test–retest reliability

Test-retest reliability is the ability of successive measurements to obtain similar results in a stable individual. The recommended time between the initial and the repeat administration of the measurement is 1 week to avoid recall and to ensure that clinically significant change has not occurred. The Thai MHQ was administered twice with a 7-day interval before treatment [36]. The intraclass correlation coefficient (ICC), which can range from 0 to 1, was > 0.7 indicating good reliability [36].

### Construct validity

Construct validity is a measure of the association between an instrument and theoretically hypotheses concerning the concepts being measured [36, 37]. The Thai version of the disabilities of the arm, shoulder and hand questionnaire (Thai DASH) [38, 39] and the Thai EQ-5D-5L [40–42] were used to evaluate the construct validity of the concepts being measured in this study. Correlation among the subscales of the Thai MHQ, Thai EQ-5D-5L and Thai DASH were measured using the Spearman’s rank correlation coefficient (r). We hypothesized that the same or related subscales should show a high correlation (Pain subscale between the Thai MHQ, the Thai DASH and the Thai EQ-5D-5L), while unrelated subscales should demonstrate a weak correlation (Aesthetics subscale in the Thai MHQ and the Pain subscale in the Thai DASH, the Aesthetics subscale in the Thai MHQ and the Mobility subscale in the Thai EQ-5D-5L) and the Thai MHQ should be strong correlated to the Thai DASH. The level of correlation was rated as low (*r* < 0.3), moderate (0.3 ≤ r ≤ 0.6), or strong (*r* > 0.6) [43].

### Thai DASH

The Thai DASH questionnaire consists of 30 self-reported items to assess symptoms and disability status. It also contains 2 optional modules, a Work and sports subscale and a Performing arts subscale [11]. The Thai version was translated by Tongprasert et al. and its psychometric properties were tested by Buntragulpoontawee et al. [38, 39]. The DASH questionnaire consists of 5 subscales: Common activities, Self-care activities, Pain symptoms, Other symptoms including numbness, joint stiffness, weakness, and sleep problems and Psychological effects. Scores range from 0 to 100, with higher scores representing greater disability.

### Thai EQ-5D-5L

The EQ-5D-5L is a two-part outcome measurement extensively used to evaluate health status [44, 45]. The first part consists of 5 subscales: Mobility, Self-care, Usual activities, Pain/discomfort and Anxiety/depression. Each subscale has five levels of severity ranging from no problems to extreme problems. The second part uses numeric scales to evaluate general health condition with scores ranging from 0 to 100, where higher scores indicate better health. The original version was translated into Thai and its psychometric properties have been evaluated [40–42]. The index value of Thai EQ-5D-5L in each patient was calculated from EQ-5D-5L Crosswalk Index Value Calculator which was available for the following countries: Denmark, France, Germany, Japan, the Netherlands, Spain, Thailand, UK, US and Zimbabwe (https://euroqol.org/eq-5d-instruments/eq-5d-5l-about/valuation-standard-value-sets/crosswalk-index-value-calculator/). We used the Thai index which ranged from − 0.452 to 1.000 [46].

Construct validity of the Thai MHQ was also investigated using confirmatory factor analysis (CFA) to evaluate the nature of and relations between latent constructs. Maximum likelihood estimation was used to evaluate the parameters. Both unidimensional construct and 6-factor-model of the Thai MHQ were analyzed. Indexes used for evaluating goodness of fit included a comparative fit index (CFI) of ≥0.95, a non-normed fit index (NFI) or Tucker-Lewis Index (TLI) ≥0.9. A root-mean-square error of approximation (RMSEA) ≤0.6 (0.08) was considered an acceptable fit [47, 48] and a c^2^/*df* < 3 [47, 48]. Modification indices were used after initial analysis, and error term correlation was used when indicated [49].

### Responsiveness

Responsiveness is the ability of a measurement to detect clinically significant changes over time [36]. Responsiveness of the Thai MHQ was evaluated by comparing the scores at baseline and at follow-up periods using the standardized response mean (SRM) and effect size (ES). SRM was calculated as the observed mean change divided by the standard deviation of the observed change while ES was evaluated as the observed mean change divided by the standard deviation of the baseline scores. SRM is the preferred value to compare paired data measurements at different time points for the same patient. We hypothesized that Thai MHQ should provide moderate to large responsiveness. SRM and ES values of 0.8, 0.5, and 0.2 were considered to be large, moderate, and small, respectively [21, 50]. The minimal important change (MIC) of Thai MHQ was evaluated using distribution methods and reported as the standard error measurement ($\mathrm{SEM}=\mathrm{Standard}\ \mathrm{deviation}\ast \sqrt{1-\mathrm{reliability}}$) [51]. The smallest detectable change (SDC) was analyzed as $1.96\times \sqrt{2}\times \mathrm{SEM}$[52].

### Floor or ceiling effects

Floor or ceiling effects were considered to be present if more than 15% of patients reported the lowest or highest possible scores [53]. If floor or ceiling effects are present, it means extreme items are missing in the lower or upper end of the scale, indicating limited content validity. Patients with the lowest or highest possible score cannot be distinguished from each other, so reliability is limited. Additionally, responsiveness is reduced since changes cannot be evaluated in these patients [36].

### Statistical analysis

For demographic data, categorical variables are reported as frequencies and percentages. Continuous variables are reported as means and standard deviations. Statistical significance was set at *P* < 0.05. In multiple comparisons, the *P*-value was adjusted using Bonferroni’s method. Following on Mundfrom et al. regarding levels of communality the ratio of the number of variables to the number of factors indicated, a minimum sample size of 200 patients was sufficient for factor analysis in this study with 37 variables and 5 factors [54]. The present study included 217 participants in the analysis.

## Results

### Translation and cross-cultural adaptation

Minor cross-cultural adaptations were modified in the activities of daily living subscale. The item “Eat with a knife/fork” was changed to “Eat with spoon and fork”. Other adaptations were made in the patient characteristics (information) section. The items “What is your ethnic background?”, “What is your racial background?” and “How long after your surgery did you return to the job you were doing before your injury” were removed. The range of family income was changed in the item “What is your approximate family income”. During field testing, 30 volunteers (15 healthy volunteers and 15 patients) completed the pre-final version. All items had an IOC >0.5, an acceptable level. The final Thai version was approved by Michigan Center for Hand Outcomes and Innovation Research.

Demographic data are shown in Table 1. Two hundred and seventeen hand/wrist injury or disorder patients were recruited. No floor or ceiling effects were found in the total scores of the Thai MHQ. The average time 114 patients took to complete the questionnaire was 12.11 min.

**Table 1:** Demographic data of patients (*n* = 217)

Age (years); mean ± SD	47 ± 17
Female; n (%)	143 (66)
Right-hand dominant; n (%)	191 (88)
Injured hand; n (%)	
Right	93 (43)
Left	97 (45)
Both	27 (12)
Diagnosis; n (%)	
Joint stiffness	7 (3)
Nerve injury	2 (1)
Wrist fracture	39 (18)
Tendon entrapment	56 (26)
Compound fracture	1 (0.5)
Joint arthritis	13 (6)
Nerve entrapment	35 (16)
Tumor	17 (8)
Tendon injury	13 (6)
Hand infection	4 (2)
Ligament injury	6 (3)
Calcific tendinitis	1 (0.5)
Joint dislocation	7 (3)
Hand fracture	8 (4)
Malunion	1 (0.5)
Soft tissue injury	4 (2)
Finger amputation	3 (1)

*SD* Standard deviation

### Internal consistency and test-retest reliability

The internal consistency of the Thai MHQ was assessed with 217 patients (Table 2). All subscales had adequate internal consistency. Most subscales (Overall hand function, Activities of daily living, Work performance, Pain, and Satisfaction with hand function demonstrated high Cronbach’s alpha values ranging from 0.94 to 0.98, while the Aesthetics subscale presented slightly lower Cronbach’s alpha values (0.79 for right, 0.80 for Left). After excluding patients who were not followed up after 1 week and patients who could not complete all the items, test-retest reliability was performed with 72 patients. The results showed all subscales had good reliability with ICC values between 0.83 and 0.95 (Table 3).

**Table 2:** Cronbach’s alpha for each subscale in the Thai MHQ (*n* = 217)

Subscale	Cronbach’s Alpha
I. Overall hand function	
Right hand	0.94
Left hand	0.94
2. Activities of daily living	
Right hand	0.97
Left hand	0.97
Both hands	0.96
3. Work performance	0.94
4. Pain	
Right hand	0.97
Left hand	0.98
5. Aesthetics	
Right hand	0.79
Left hand	0.80
6. Satisfaction with hand function	
Right hand	0.95
Left hand	0.96

*MHQ* Michigan Hand Questionnaire

**Table 3:** Test-retest reliability of the Thai MHQ (*n* = 72)

Subscale	Baseline	Retest	ICC	95% confidence Interval	
	Mean ± SD	Mean ± SD		Lower bound	Upper bound
Overall hand function	64.90 ± 19.79	65.17 ± 23.07	0.93	0.88	0.95
Activities of daily living	75.36 ± 23.42	74.72 ± 25.82	0.87	0.79	0.92
Work performance	54.58 ± 30.10	58.06 ± 32.18	0.83	0.73	0.90
Pain	40.03 ± 28.86	36.18 ± 29.31	0.95	0.92	0.97
Aesthetics	60.59 ± 25.71	61.20 ± 24.23	0.87	0.79	0.92
Satisfaction with hand function	55.82 ± 26.36	55.84 ± 26.45	0.90	0.84	0.94
Thai MHQ	61.87 ± 21.08	63.13 ± 22.62	0.95	0.91	0.97

The distribution of patients based on the diagnosis was 17 wrist fracture (23.6%), 4 hand fracture (5.6%), 16 tendon entrapment (22.2%), 7 joint arthritis (9.7%), 11 nerve entrapment (15.3%), 5 ligament injury (6.9%), 8 tendon injury (11.1%), 1 malunion (1.4%) and 3 tumor (4.2%)

*ICC* Intraclass correlation coefficient, *MHQ* Michigan Hand Questionnaire, *SD* Standard deviation

### Construct validity

Evaluation of the construct validity of the Thai MHQ was conducted with 217 patients. The correlation of subscales for the Thai MHQ, the Thai DASH and the Thai EQ-5D-5L was compared (Table 4). In related dimensions, the assessment of the Pain subscale between the Thai MHQ, the Thai DASH and the Thai EQ-5D-5L found a relatively strong correlation between the Thai MHQ and the Thai DASH (*r* = 0.58, *P* < 0.0001) as well as between the Thai MHQ and the Thai EQ-5D-5L (*r* = 0.59, *P* < 0.0001). A strong correlation (*r* = 0.77, *P* < 0.0001) was demonstrated between the Activities of daily living in the Thai MHQ and the Common activities in the Thai DASH, indicating convergent validity. Among unrelated dimensions, there were the weak negative correlations between the Aesthetics subscale in the Thai MHQ and the Pain subscale in the Thai DASH (*r* = − 0.20, *P* = 0.003) and also between the Aesthetics subscale in the Thai MHQ and the Mobility subscale in the Thai EQ-5D-5L (*r* = − 0.13, *P* = 0.05) indicating discriminant validity. There were the strong correlations between Thai MHQ and Thai DASH (*r* = − 0.79, *P* < 0.0001) and between Thai MHQ and Thai EQ-5D-5L (*r* = 0.63, *P* < 0.0001).

**Table 4:** Spearman’s correlation for each subscale among the Thai MHQ, Thai DASH and Thai EQ-5D-5L (*n* = 217)

**(4A) Thai DASH**						
**Thai MHQ**	**Common activities**	**Self-care activities**	**Pain**	**Other symptoms**	**Psychological effects**	**Total score**
**Overall hand function**	− 0.58*	− 0.53*	− 0.40*	− 0.33*	− 0.33*	−0.59*
**Activities of daily living**	−0.77*	− 0.72*	− 0.49*	− 0.41*	− 0.38*	− 0.78*
**Work performance**	− 0.58*	− 0.49*	− 0.44*	− 0.35*	− 0.32*	−0.58*
**Pain**	0.57*	0.52*	0.58*	0.54*	0.43*	0.62*
**Aesthetics**	−0.38*	−0.33*	−0.20	− 0.32*	−0.37*	− 0.40*
**Satisfaction with hand function**	−0.59*	− 0.50*	−0.51*	− 0.47*	−0.37*	− 0.62*
**Total score**	−0.77*	− 0.68*	−0.58*	− 0.52*	−0.51*	− 0.79*
**(4B) Thai EQ-5D-5L**						
**Thai MHQ**	**Mobility**	**Usual activities**	**Self-care activities**	**Pain**	**Anxiety/Depression**	**Total score**
**Overall hand function**	−0.12	−0.44*	−0.37*	− 0.43*	−0.20	0.45*
**Activities of daily living**	−0.19	−0.53*	− 0.55*	−0.50*	− 0.33*	0.60*
**Work performance**	−0.15	−0.47*	− 0.33*	−0.41*	− 0.25	0.43*
**Pain**	0.11	0.48*	0.35*	0.59*	0.33*	−0.51*
**Aesthetics**	−0.13	−0.30*	− 0.31*	−0.28*	− 0.30*	0.36*
**Satisfaction with** **hand function**	−0.09	−0.50*	− 0.34*	−0.53*	− 0.38*	0.48*
**Total score**	−0.17	−0.61*	− 0.50*	−0.59*	− 0.40*	0.63*

*Statistically significant (*p* < 0.0025) adjusted for multiple comparison using Bonferroni’s method

*DASH* Disabilities of Arm, Shoulder and Hand, *MHQ* Michigan Hand Questionnaire

*CFA*

The unidimensional model showed an acceptable fit to the data with a CFI of 0.985, TLI of 0.983, RMSEA of 0.080 (90%C.I., 0.073–0.087), SRMR of 0.067, and a χ^2^ = 891.834, *df* = 374 (*p* < 0.001). However, the 6-factor solution fitted the data best with the following statistics: CFI was 0.995, TLI was 0.994, RMSEA was 0.047 (90%C.I., 0.039–0.055), SRMR was 0.041, and a χ^2^ = 570.062, *df* = 385 (*p* < 0.001). The fit statistics for all models were based on the fact that the error terms of the indicators within the same subscale were correlated.

### Responsiveness

Fifty patients continued with followed-up after receiving initial treatment and completed all items in the Thai MHQ questionnaire both before treatment and at follow-up. The mean time to follow-up was 52 days. The three most common treatments were steroid injection (32%), surgical fixation (26%) and surgical release (20%). The SRM and ES of the Thai MHQ were 0.78 and 0.69 respectively indicating relatively large responsiveness (Table 5). SRM and ES of Thai MHQ based on each treatment were demonstrated in Table 6. The MIC of Thai MHQ using distribution methods (SEM) was 5.2 while the SDC was 14.4.

**Table 5:** Standardized response mean (SRM) and effect size (ES) of each questionnaire (*n* = 50)

	Baseline Mean ± SD	Follow-up Mean ± SD	Mean difference	SD of change	SD of baseline	SRM	ES
Thai MHQ	52.12 ± 19.91	65.93 ± 21.03	−13.81	17.63	19.91	−0.78	− 0.69
Thai DASH	53.21 ± 17.17	40.49 ± 17.11	12.72	15.04	17.17	0.85	0.74

Time to follow-up = 52 ± 25 days

*DASH* Disabilities of Arm, Shoulder and Hand, *MHQ* Michigan Hand Questionnaire, *ES* Effect size, *SD* Standard deviation, *SRM* Standardized response mean

**Table 6:** Standardized response mean (SRM) and effect size (ES) of Thai MHQ based on each treatment (*n* = 50)

Treatment n (%)	Time to follow-up Mean (days) ± SD	Mean difference	SD of change	SD of baseline	SRM	ES
Surgical fixation = 13 (26%)	81 ± 17	−18.58	22.68	23.63	−0.82	− 0.79
Steroid injection = 15 (32%)	39 ± 14	− 10.87	17.15	13.11	− 0.63	−0.83
Surgical release = 11 (20%)	32 ± 13	−12.25	13.18	12.12	−0.92	−1.01
Immobilization with splint = 5 (10%)	50 ± 16	− 17.64	18.93	24.04	− 0.93	− 0.73
Surgical repair = 6 (12%)	61 ± 28	−10.47	15.28	30.61	− 0.69	−0.34

*MHQ* Michigan Hand Questionnaire, *ES* Effect size, *SD* Standard deviation, *SRM* Standardized response mean

## Discussion

In the process of translation and cross-cultural adaptation, previous studies have demonstrated no major linguistic or cultural discrepancies [23–26, 28–32, 55–57]. In the Thai MHQ, a minor change was made in the item “Eat with a knife/fork” to be “Eat with spoon and fork” because most Thai people use a spoon as the primary eating utensil while a fork is used to manipulate the food. This is in concordance with the Korean and Japanese versions which had specific cultural eating modifications. Koreans use a spoon and chopsticks, while Japanese eat a meal with chopsticks and a rice bowl [25, 26]. However, the previous Thai version still used “Eat with a knife and fork” as the original English version [32]. Another different item between the current and the previous Thai versions was one of the items to evaluate the ability to do your routine work. This version used “How often did you have to reduce your work quality due to problems with your hands/wrists?” while the previous version provided the meaning as “How often did you have to reduce the steps of working in order to do your work easily due to problems with your hands/wrists?”. In the information part, both Thai versions removed the items which involved ethnic or racial background since the majority of the population in Thailand identify themselves as Thai. The range of income was also changed in both Thai versions. However, the previous Thai version added more information in medical expense reimbursement plan [32].

In the evaluation of internal consistency, most subscales of The Thai MHQ had Cronbach alpha values higher than 0.8 (range 0.94 to 0.98), although the Aesthetics subscale was slightly lower (0.79 for right and 0.80 for left). The internal consistency of this subscale is comparable to the Korean (0.79, 0.80), Turkish (0.76, 0.79), Canadian French (0.83, 0.79) and previous Thai versions (0.48) [23, 25, 32, 58]. The reason for the lower values for the Aesthetics subscale could be that the first item of this subscale is positive (satisfaction with appearance) while the other items are negative, i.e., level of discomfort, depression and feeling that appearance interfered with normal social activities. Nevertheless, the original and other versions have demonstrated high internal consistency (Cronbach alpha values = 0.81–0.97) [10, 24, 26, 31, 57].

Exploration of the test-retest reliability of the Thai MHQ showed good to excellent reliability in all subscales (ICC = 0.83–0.95), results comparable to the original MHQ and other versions (ICC range 0.73 to 0.99) [10, 23–25, 28, 29, 32, 55, 57, 58]. Only the Japanese version showed a slightly lower ICC (0.68) in the left Aesthetics subscales because the majority of the cohort had no injury to the left hand [26]. The Finnish version similarly presented a low ICC (0.66) in the left Aesthetics subscales; the authors suggested the first item might have led to patients’ misunderstanding and then giving contradictory answers as described in the previous paragraph [31].

Construct validity of Thai MHQ was analyzed using 2 methods: correlation of each of the subscales for common outcome measurements (Thai DASH, Thai EQ-5D-5L) and exploratory factor analysis (EFA). In comparing subscales between the Thai MHQ and other instruments, a strong correlation was found between the Activities of daily living subscale in the Thai MHQ and the Common activities subscale in the Thai DASH (*r* = − 0.77). This correlation could be due to the similarity of questions in both subscales. Among unrelated subscales, a weak correlation was revealed when comparing each subscale of the Thai MHQ and the Mobility subscale of the Thai EQ-5D-5L (*r* < 0.3) (Table 4). These results are in concordance with our hypotheses described in the Methods section. Although there were the specific subscales in the Thai EQ-5D-5L (Mobility) and the Thai MHQ (Aesthetics). We still found the moderate to strong correlations between other subscales of the Thai MHQ and the Thai EQ-5D-5L. Therefore, the total score of the Thai MHQ and the Thai EQ-5D-5L demonstrated the strong correlation (Table 4).

Our CFA findings were consistent with the original version, supporting the 6 hypothesized model [10]. However, the unidimensional model also showed acceptable fit indicating that the sum score of the Thai MHQ can be used to represent the overall function of the hand. In addition, our CFA results support a previous study that found the Aesthetics subscale had a weak correlation with other subscales. The fit statistics of the models were much improved when the Aesthetics subscale was removed [59].

The responsiveness of the Thai MHQ demonstrated a relatively large standardized response mean (SRM = 0.78) which was slightly lower than the SRM of the Thai DASH for the same cohort (0.85) (Table 5). This result might be due to the more subscale in Thai MHQ (Aesthetics) compared to Thai DASH. The level of responsiveness varied with the type of disorder, the type of treatment and the follow-up period (Table 6). As a result, the responsiveness of each version varied. The original version demonstrated a large SRM (0.8) in patients who were diagnosed with carpal tunnel syndrome and who underwent surgical release with a 6-month postoperative follow-up [22]. The Dutch version had a moderate SRM (− 0.72) with 28 rheumatoid arthritis patients and a 3-month follow-up; the majority of those patients received conservative treatment [56]. The Dutch version was also used to evaluate patients with systemic sclerosis. That evaluation found a moderate SRM (− 0.74) with 25 patients who received a multidisciplinary team care program and a 12-week follow-up [60]. The German MHQ had an SRM of 1.7 in 35 patients with trapeziometacarpal osteoarthritis who underwent surgical treatment and had a 1-year follow-up [55]. The SRM of the Korean version for each subscale was moderate to large (0.6–1) in 37 carpal tunnel syndrome patients who were evaluated at 6 months after surgery [61]. The MIC values could be interpreted based on specific diagnoses, treatments and also methodologic approaches [51]. The previous literatures reported MIC of MHQ ranged from 4.9 to 23 [14, 51].

There are some limitations in the present study. First, responsiveness to treatment for each diagnosis was not included because our aim was to focus on generalized hand/wrist injuries or disorders. Further studies could be conducted to evaluate the responsiveness with specific diagnoses, treatments and follow-up periods. Second, in the test-retest reliability and responsiveness assessments, patients who were not followed up or who did not complete the entire questionnaire were excluded, although the number of patients remaining was still comparable to previous studies [23–26, 28, 29, 31, 32, 55, 57, 58]. Finally, the Thai MHQ should be further evaluated according to item response theory, e.g., the Rasch measurement model, to provide additional information on both persons and item calibration.

## Conclusions

The Thai MHQ provides adequate internal consistency in all subscales as well as good construct validity and reliability for assessing Thai patients with wrist/hand injuries or disorders and provides a relatively large standardized response mean at 2 months after treatment. The Thai MHQ is one of the standard patient-rated outcome measurements available for clinicians and researchers to use in evaluating symptoms and functions of the hand/wrist in both clinical healthcare and in medical research.

## Data Availability

The datasets used and/or analyzed during the current study are available from the corresponding author on reasonable request.

## Abbreviations

CFA: Confirmatory factor analysis
CFI: Comparative fit index
COSMIN: COnsensus-based Standards for the selection of health Measurement Instruments
DASH: Disabilities of Arm, Shoulder and Hand
ES: Effect size
ICC: Intraclass correlation coefficient
MIC: Minimal important change
MHQ: Michigan Hand Questionnaire
NFI: Non-normed fit index
r: Spearman’s rank correlation coefficient
RMSEA: Root-mean-square error of approximation
SDC: Smallest detectable change
SEM: Standard error measurement
SRM: Standardized response mean
STROBE: Strengthening the Reporting of Observational Studies in Epidemiology
TLI: Tucker-Lewis Index
WHO ICF: World Health Organization International Classification on Functioning Disability and Health
